# Review of Ethanol Intoxication Sensing Technologies and Techniques

**DOI:** 10.3390/s22186819

**Published:** 2022-09-09

**Authors:** Szymon Paprocki, Meha Qassem, Panicos A Kyriacou

**Affiliations:** Research Centre for Biomedical Engineering, School of Science and Technology, University of London, Northampton Square, London EC1V 0HB, UK

**Keywords:** ethanol, intoxication, sensors, devices, algorithms

## Abstract

The field of alcohol intoxication sensing is over 100 years old, spanning the fields of medicine, chemistry, and computer science, aiming to produce the most effective and accurate methods of quantifying intoxication levels. This review presents the development and the current state of alcohol intoxication quantifying devices and techniques, separated into six major categories: estimates, breath alcohol devices, bodily fluid testing, transdermal sensors, mathematical algorithms, and optical techniques. Each of these categories was researched by analyzing their respective performances and drawbacks. We found that the major developments in monitoring ethanol intoxication levels aim at noninvasive transdermal/optical methods for personal monitoring. Many of the “categories” of ethanol intoxication systems overlap with each other with to a varying extent, hence the division of categories is based only on the principal operation of the techniques described in this review. In summary, the gold-standard method for measuring blood ethanol levels is through gas chromatography. Early estimation methods based on mathematical equations are largely popular in forensic fields. Breath alcohol devices are the most common type of alcohol sensors on the market and are generally implemented in law enforcement. Transdermal sensors vary largely in their sensing methodologies, but they mostly follow the principle of electrical sensing or enzymatic reaction rate. Optical devices and methodologies perform well, with some cases outperforming breath alcohol devices in terms of the precision of measurement. Other estimation algorithms consider multimodal approaches and should not be considered alcohol sensing devices, but rather as prospective measurement of the intoxication influence. This review found 38 unique technologies and techniques for measuring alcohol intoxication, which is testament to the acute interest in the innovation of noninvasive technologies for assessing intoxication.

## 1. Introduction

Ethanol consumption is a major component of social life in the Western world. Ranging from casual drinking or as a part of celebration to more extreme binge drinking or alcohol dependence/alcoholism, often referred to as alcohol use disorder (AUD) [1], alcohol consumption has also been associated with the development of several types of cancer [2]. With high frequency of consumption of alcoholic beverages and the corresponding effects of alcohol intoxication on the body and behavior of individuals, a necessity for quantification of intoxication has become an important part in assessing the state of an individual. Driving under the influence (DUI) of alcohol in the UK is related to an estimated 13% of all fatal road accidents and is a major cause of death for males between 15 and 59 years of age [3]. In an effort to prevent these tragedies, several methods have been developed to estimate intoxication levels spanning many fields, such as biochemistry, physiology, photonics, electronics, image analysis, and artificial intelligence.

Alcohol intoxication is a standardized metric denoted by blood alcohol concentration (BAC) only and not the effect it has on an individual, thus not accounting for tolerance resulting from regular exposure to ethanol. Although similar symptoms of intoxication can be seen amongst individuals, the influence of alcohol tolerance remains a poorly explored phenomenon in the context of the wider population. The BAC level corresponds to the weight of ethanol in milligrams per 100 mL of blood. The level of intoxication is positively correlated with the amount of ethanol in the bloodstream, with the high end of ethanol intoxication at 0.5% (500 mg/dL) and with levels as low as 0.35% (350 mg/dL) being linked to death or serious harm to the individual or those around them [4]. Regular consumption of excessive amounts of alcohol is also associated with the development of liver disease and increased blood pressure, making those individuals more susceptible to health complications in the future [5]. The legal drinking limit for driving in the UK is 80 mg per 100 mL of blood, equivalent to a BAC of 0.08, which can be categorized as one of the higher levels of alcohol permissible to drive, whilst many European countries and Middle Eastern countries allow for a very low level of intoxication (BAC 0.02) or prohibit driving under the influence of alcohol altogether under a “zero tolerance” policy. The territory with the highest permissible level of BAC is the Cayman Islands allowing a BAC level of 0.10. Besides DUI, alcohol consumption can also be linked to crimes, such as theft and criminal damage, and in such circumstances alcohol serves as a catalyst for antisocial behavior and violent crime [6]. Alcohol consumption puts a significant burden on public services. Combining the costs of dealing with alcohol-related crime, loss of productivity through unemployment and sickness, and the cost and burden on the National Health Service (NHS), the cost of alcohol on society is estimated to be GBP21 billion per year [7], although the real figure is thought to be even higher. Reviews on the subject of economic impacts of alcohol consumption express the cost figure as percentage of gross domestic product (GDP) ranging between 0.45% and 5.44% annually [8].

Short-term influences of alcohol intoxication, however, do not carry such damaging consequences, yet they are not without harm. Acute intoxication can have damaging effects on people diagnosed with cancer or currently taking antibiotics. The reaction of ethanol in the liver can trigger inflammation and damage the liver of the user. Other cases where acute consumption poses a risk of damage is particularly seen amongst people who are suicidal, increasing the risk of taking their life. [9]. Ethanol affects the body by influencing the central nervous system through the inhibition of gamma-aminobutyric acid (GABA) receptors [10]. This results in reduced cognitive ability, slurred speech, loss of balance, and reduced social inhibition. Long-term consumption can lead to alcohol use disorder (AUD). Neuroscience researchers have also found a correlation between neuron activity and metabolites of ethanol, such as acetic acid [11]. This correlation may suggest that other chemical imbalances contribute to intoxication effects. The effects of acetic acid on the nervous system have not been studied in as much depth as ethanol, and could potentially prove to be an important component for quantifying intoxication influences or relating to the addictive properties of alcohol consumption. Globally, excessive consumption of alcohol leads to AUDs and addictions, with an estimated 586,780 sufferers of AUD just in the UK and only 18% receiving treatment [12].

With so many problems associated with alcohol consumption, methods of estimating alcohol intoxication were reported in medical literature as early as 1920 by Widmark [13]. With further development in technology and chemical analytics, several methods, such as gas chromatography, became available for measuring intoxication levels in a variety of bodily fluids. Similarly, this development in technology and analytical techniques gave rise to the most notable alcohol intoxication measuring device, the Breathalyzer™, a breath alcohol content (BrAC) measuring device. This method allows for remote BAC testing, particularly for traffic safety, without the need to send blood samples for laboratory analysis [14].

Currently, sensing alcohol intoxication techniques include devices of various natures that take advantage of machine learning, optical spectroscopy, and biochemical sensing methods. This review aims to comprehensibly explore all available literature on quantitative and qualitative assessment of alcohol intoxication, as well as observing the evolution of approaches and methodologies for collecting these measurements and exploring the current gaps in the field of in vivo sensing of alcohol intoxication.

## 2. Toxicology of Intoxication

Intoxication can be defined as loss of control over actions or behavior changes under the influence of a drug. Intoxication due to ethanol can be divided into three main parts: initial take-up, the peak, and the decay stage. This can be illustrated by studies performed on human volunteers to investigate the changes of alcohol in their blood over time [15]. The initial uptake of ethanol causes the blood alcohol concentration (BAC) to raise rapidly, reaching peak intoxication between 30 to 60 min, although that number is heavily dependent on the dosage. After that, the peak BAC levels begin to decay, reaching zero between six to eight hours after initial consumption. This, however, is also dependent upon the volume of ethanol consumed. The standard unit of measurement of alcohol intoxication is not internationally agreed upon, with variation in the order of magnitude of measurement as well as the numerical systems used. In the medical literature, the consensus on measurement is to use BAC as volume of pure ethanol per 100 mL of blood, varying from 0 to 0.5, representative of concentration levels between 0 and 500 mg/dL.

Considering the uptake of ethanol, this period is characteristic of euphoric behavior, including laughter, social inhibition, and generally increased well-being due to the release of hormones, such as serotonin. At the peak of intoxication, these effects begin to slowly fade away, due to decreasing levels of ethanol in the body. The roll-off stage is associated with increased tiredness and depression [16]. The primary influence of ethanol intoxication originates in the central nervous system through the inhibition of GABA receptors. Alcohol molecules inhibit the active site of GABA receptors, resulting in reduced cognitive function and decreased spatial awareness. Alcohol also contributes to the production of serotonin, resulting in a relaxed state of the consumer [17], hence enacting on the reward system of the brain. With time, these effects wear off, depending on several factors, such as age, sex, and body weight. The literature correlates sex with an aspect of varied breakdown of ethanol, possibly explained by the lower resting metabolic rate in women [18]. Tolerance is also a factor when considering the decay of ethanol in the blood, as has been demonstrated by people with AUD that can metabolize ethanol at a faster rate than occasional drinkers [19]. In the body, alcohol is subject to many chemical reactions, specifically those involved in its breakdown. A group of enzymes responsible for ethanol breakdown are known as alcohol dehydrogenases. These enzymes are responsible for breaking down alcohol into acetaldehydes and subsequently acetic acid. These waste products are dealt with in the body by means of various other enzymes. Specifically, acetic acid is a subject in the acid cycle for neutralization. It is key to highlight that high concentration of these acids can lead to acidosis, a symptom of alcohol poisoning, requiring medical attention in severe cases [20]. Besides inhibiting GABA receptors and being broken down by enzymes, alcohol also influences the function of the cardiovascular and pulmonary systems. Primarily, the impact of ethanol on the blood vessels extends to the function of relaxation by vasodilation. It is key to note that although alcohol relaxes the blood vessels, this is only seen for small doses of alcohol. This is also a contributing factor to the beneficial health impacts of alcohol. However, act as exclusively limited to small doses of alcohol. At higher levels of BAC, it begins to take on a pressor, restricting the blood vessels [21]. This once again can be attributed to the acids produced through the metabolic breakdown of ethanol, although the true origin of this effect is not clear.

Alcohol affects a number of systems in the body, resulting in an intoxicated state. As mentioned previously, these effects manifest themselves in bodily organs, such as the heart, lungs, liver, and brain. However, these effects are short-lived and fade away after time. On the other hand, long-term consumption of excessive amounts of alcohol can contribute to a multitude of diseases, both physical and mental. Amongst them are the mental illness associated with dependence or addiction to alcohol. The root causes of these diseases are mostly unexplored in terms of explaining the susceptibility to developing an alcohol addiction [22]. Some research suggests that both genetic and environmental factors play a role in the development of AUD [23]. AUD is often characterized by large and frequent consumption of alcohol, as well as by withdrawal symptoms, some of which include tachycardia, tremors, sweating, delirium, seizures, insomnia, and anxiety [24]. Several treatments exist to help recovering people with AUD [25,26]. Regular and uncontrolled consumption of alcohol can lead to an AUD, which, if untreated, can become a gateway for development of more serious health problems, some of which are fatal. Cardiac health is significantly impacted by excessive and regular consumption of alcohol. Amongst the long-term effects of alcohol consumption are alcoholic cardiomyopathy (change of shape of the heart), high blood pressure, myocardial infarction (heart attack), arrythmias (irregular heart rhythm), fatal cardiac arrest, and stroke [27]. The association between heavy alcohol use and cardiovascular disease (CVD) is unclear. Discussion on this topic focuses on alcohol’s effect on the atherosclerotic process (hardening of blood vessels) in vessels and the toxic damage to the myocardium [28]. As the main site of alcohol metabolism, the liver experiences the most damage, although much of that is mitigated by its regenerative properties [29]. However, even that is not enough to prevent the tissue damage caused by excessive and prolonged consumption of alcohol. Chronic and excessive alcohol consumption results in the formation of hepatic lesions on the liver, including steatosis (deposition of fat in hepatocytes), hepatitis (inflammatory type of liver injury), and fibrosis (tissue scarring) [30]. Continuous damage to the tissue of the liver and the formation of scar tissue contributes to and increase the risk of developing liver cancer, a very prominent disease amongst heavy alcohol users. AUD and heart and liver damage are just a few of the many pathologies that can be attributed to excessive consumption of alcohol [31,32]. Alcohol-related disease is a big burden on the health system.

## 3. Technologies and Devices

The literature review of ethanol intoxication sensors yielded several results encompassing different aspects of alcohol intoxication, i.e., behavioral, physiological, and chemical changes in the individual’s body. All the methods were categorized into six main sections: pharmacokinetic estimates, breath-sample testing, bodily fluids, physiological changes, transdermal, and optical spectroscopy. The findings of the review and all the devices and techniques considered are summarized in Table 1.

As seen from Table 1, the field of alcohol intoxication sensing is filled with innovative methods of analyzing factors of intoxication, not exclusively changes in the concentration of ethanol biomarkers but also tracking physiological changes occurring during an intoxication episode. A great deal of attention in the literature is given to laboratory methodologies of detecting ethanol and its biomarkers through forensic analysis. These methods focus on establishing not only the intoxication level itself but also the exposure level, such as that seen in hair or nail samples, as opposed to gas chromatography blood testing. Several publications showcase the latest developments and ideas, for which the trial and experimental data are publicly available. Table 2 and Table 3 summarize these findings.

### 3.1. Early Estimates of BAC

The earliest methods of quantifying the level of alcohol in blood were introduced by Erik Widmark in 1918 [13] by a method based on Nicloux’s oxidation separation, with an introduction of the S-shaped capillary tube for blood collection from the finger or the earlobe. The ethanol content was measured by pouring the collected blood into a glass cup affixed to the neck of the flask. The ethanol would then separate from the blood by diffusion. The results of this experiment were reported as mass concentration unit, giving rise to one of the first units of alcohol blood contents—the promille. The final level of ethanol was then determined by titration.

(1) $$EBAC=\frac{A}{Wrt}-\beta T$$

The equation introduced by Widmark and Tandberg in 1924 [33] aimed to estimate the levels of alcohol in the blood through analysis of the behavior of ethanol as it was metabolized in the body. In their work, they proposed a mathematical formula to determine ethanol levels in the blood of a subject based on the following parameters: amount of ethanol consumed, body weight, alcohol elimination rate (*β*), distribution of water in the body (*r*), and the consumption start time. This correlation is outlined in Equation (1). It is key to note that the values of constants *β* and *r* are subject to variation across individuals, most noticeably between sexes [34].

This method of calculating the estimated level of BAC has been at the forefront of forensic examinations and contributed significantly to the field of ethanol toxicology [35]. Widmark and Tandberg also developed similar models for levels of methanol and acetone [33].

For its time, the *EBAC* equation provided a quick and easy to use method for determining ethanol levels in the body; however it has several shortcomings, mainly those associated with the values of *β* and *r*. Since these values vary considerably among individuals, the values used for generic applications of *EBAC* calculations do not often account for this natural variance. For this reason, Widmark developed variants of this equation to account for natural variations between men and women, thereby measuring the uncertainty of the result. As a result, the uncertainty of the *EBAC* is around ± 20% [36]. This high uncertainty is the product of the assumption of general values for *β* and *r*, as well as other parameters in the *EBAC* equation.

Although significant in defining the metabolism of ethanol, the value of *β* is a subject of large variation and thus not truly representative of the general population. As a result, further work in this area aimed at relating levels of ethanol in the blood to those in air exhaled after the consumption of alcohol.

#### 3.1.1. Advantages

- The Widmark equation is a very useful tool for estimation of blood alcohol levels in the absence of testing equipment.
- The estimations consider the sex and the elimination rate of ethanol, accounting for some of the most important factors in ethanol metabolism.
- The Widmark flask is the first proven method of measuring ethanol levels in the blood.

#### 3.1.2. Disadvantages

- The Widmark flask requires blood samples, making it an invasive procedure.
- The Widmark equation is not completely representative of true intoxication levels.
- The Widmark equation requires use of tables and knowledge of time consumption.

### 3.2. Breath Alcohol Devices

The earliest mentions relating BAC to breath alcohol concentration (BrAC) date back to 1874, by Baldwin [37], with observations that showed the presence of ethanol in the subject’s breath after consumption of an alcoholic beverage. Later research in the field by Bogen in 1927 demonstrated a method for quantification of BrAC and using it to estimate BAC [38]. The first widely available commercial product for quantifying BAC, the Breathalyzer™, developed by Borkenstein [39], correlated the concentration of ethanol in the bloodstream to the amount of ethanol exhaled via measurement of coulometric changes in the reaction of ethanol and acid dichromate. The idea of using breath samples to estimate levels of ethanol in the bloodstream was implemented in law enforcement for prevention of driving under the influence and traffic safety.

The scope of development of breath alcohol devices is broad and encompasses many areas of chemical analysis to determine the level of ethanol, subsequently giving rise to various devices measuring BrAC. Regardless of the variations in the devices used to measure BrAC, one feature remains unchanged: the method of calibration. Most BrAC devices consider a calibrating ratio of 2100:1 for the alveolar air and ethanol volume, such that 2100 mL of alveolar air is equivalent to 1 mL of ethanol in a breath sample [40]. This approximation is the major criticism of BrAC devices. Naturally, the above ratio can vary between individuals by as much as 1448–3818 [41]. The value of 2100 has been taken as an average value for alveolar lung volume and is used in the calibration of BrAC devices. Depending on the country of use, the ratio used may vary. As a result, BrAC device readings are heavily dependent on lung volume with a bias against individuals with lower lung sizes [42]. It has also been shown that BrAC levels depend on the breath effort [42].

BrAC devices may be divided into four main categories, based on the ethanol detection principle they employ. Photovoltaic assays measure the color change of a liquid experiencing a redox reaction, usually from red/orange to green, when ethanol is detected. The degree of color change is measured and translated into an output of BrAC level and converted into BAC [39]. Infrared spectroscopy measures the spectral change in the breath sample by passing a beam of NIR radiation though the sample, with high specificity for ethanol [43]. Fuel cell BrAC devices measure the oxidation of ethanol into acetaldehyde on an electrode cell. The output of the current is measured across the cells, which is proportional to the level of oxidation and in turn, the amount of ethanol in the breath sample [44]. Fuel-cell alcohol devices come in much smaller form and can be made portable. This feature, amongst others, makes it the most popular type of BrAC device [45]. Semiconductor-based BrAC devices operate on the change in conductance of metal oxide layers in the presence of ethanol, which act as a reducing agent [46]. Semiconductor BrAC devices are still in the early stages of development; however, they present an opportunity for further miniaturization of portable alcohol testing devices.

**Figure 1:**
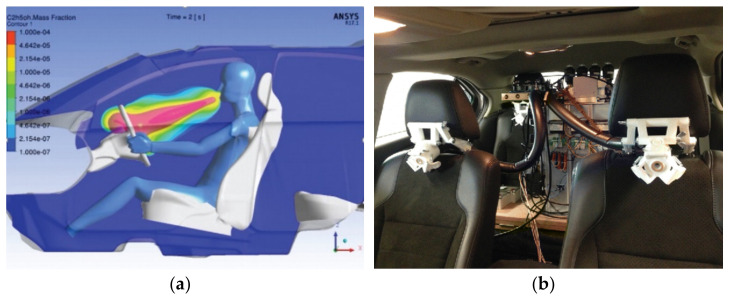
Autoliv breath alcohol sensing system. (**a**) Simulated view of the gas path from the driver to the sensor on the steering wheel. (**b**) Prototype placement of the sensors in the car. [47].

An innovative sensor developed by Ljungblad et al. [47] proposes a method of passively measuring the content of ethanol in the breath of the driver. The mechanism of the sensor can detect the location of the exhaled ethanol by measuring the concentration of CO_2_ exhaled by means of infrared spectroscopy (IRS). This feature aims to minimize false positives originating from spillage of ethanol-containing beverages or cleaning products. This technique is designed to be implemented in a vehicle, where each of the passengers can be monitored passively throughout the duration of a car ride. This implementation, however, shares many of the same shortcomings and sources of error as any other traditional BrAC devices [48]. The sensor is still in the stages of development, and as noted by the author, the system requires improvements in robustness and resolution. A simulated view of the system operation and the in-vehicle prototype are shown in Figure 1 below.

In general, BrAC devices are convenient and effective for estimating BAC by considering an associated measure of BrAC. It is key to note that BrAC devices are prone to erroneous readings and can only be used to estimate the level of ethanol in a subject’s blood. Besides the error associated with the calibration of these devices, the environmental conditions under which the devices operate must also be considered, specifically the temperature, as demonstrated by Legge [49]. Legge also cited that those errors of measurement are not only impacted by the environmental factors during the test but also the equilibrium of the ethanol in the respiratory tract, relative to individual breathing patterns. This was later confirmed by Jones [50], who showed that holding the breath increases the concentration of expired ethanol by up to 18%, whilst hyperventilation can decrease it by up to 12%. Therefore, for BrAC, system corrections are applied in numerous cases to achieve the most accurate results, many of which, as suggested by Dubowski [48], should include subtraction of 0.055% from all results. For the time being, BrAC devices are here to stay and still prove useful as a deterrent to drunk driving, as well as assisting in the serving of justice.

#### 3.2.1. Advantages

- Proven to be effective in law enforcement, prevention of DUI
- Nonintrusive procedure
- Results generated within seconds of the reading
- Passive measurements of an enclosed space possible
- Possibility to incorporate BrAC into vehicles, for example, the Interlock system
- Portable, small form factor, light, battery powered.

#### 3.2.2. Disadvantages

- A large selection of devices available in retail outlets, many of varying quality
- Well-performing breathalyzers are very expensive and require laboratory calibration.
- BrAC devices apply an outdated conversion ratio of 2100:1, which has been proven to vary between sex and lung size.
- In cases of more accurate BrAC devices, such as the Intoxilyzer. the form factor is increased and requires a permanent allocation.
- Testing requires replaceable parts.

### 3.3. Bodily Fluid Testing

Amongst alcohol testing techniques, the bodily fluids of blood, urine, and saliva yield large variations, both in technique implementations and the results obtained by each method. Hence, each finds its application in a different field of testing.

Bodily fluid testing concerns the measurement of ethanol concentration in the blood, urine, and saliva by measuring it directly from the blood itself as the gold standard of such measurements. In order to determine the concentration of ethanol in the sample, the most common technique used is gas chromatography (GC) or headspace GC, which measure the presence of exact levels of ethanol in a sample through separation of components in a gas state [51]. These tests can be performed on whole blood as well as plasma and serum, with the highest possible accuracy [52]. However, it is not the only technique that can be used to determine the level of ethanol in a blood sample. Alternative methods include oxidimetric methods [53]: separation of ethanol from blood via diffusion, distillation, or aeration and reacting ethanol with oxidizing agents (dichromate or permanganate), where the residual agent determines the level of ethanol. Other techniques include the use of enzymes to determine the level of ethanol in a blood sample [54]. Such methods utilize ADH and NADH to oxidize ethanol into acetaldehyde and use enzyme kinetics to estimate the level of ethanol present in the sample.

It is key to note that blood samples must be handled with care and in many cases adhere to rigorous standards of storage and handling [55]. These include and are not limited to airtight sealing of the tubes and mixing in additives, such as anticlotting agents and preservatives, to prevent pathogen growth.

Blood ethanol testing also comes with its own array of strengths and shortcomings, which determine its viability in ethanol testing. As the most direct method of measuring ethanol levels, the results obtained are considered highly accurate and representative; however, one must consider the site of blood-sample collection, since arterial blood has higher concentrations of ethanol, but venous blood samples are easier to acquire [56]. Blood testing also requires a small sample to obtain measurements, as little as 2.5 µL of plasma [57]. However, blood sampling is an invasive procedure, which makes it difficult to be implemented in a standardized testing environment, such as a roadside. Besides this, samples often must be sent off to external laboratories, due to the cost of equipment required for testing, more specifically the gas chromatograph. Therefore, blood ethanol testing finds most of its applications in forensics and research.

Besides blood testing, BAC levels can also be estimated from urine samples. However, this method comes with an entire range of shortcomings and is thus not useful for accurate determination of ethanol levels in the subject’s bloodstream. The greatest error associated with urine ethanol testing comes from the behavior of ethanol in the body, more specifically the changes in ethanol level during the absorption and elimination stages [19]. During this time, the concentration of ethanol can change drastically. Therefore, urine samples are more commonly used to determine the presence of ethanol in a sample, but not quantify the level of intoxication. This is because urine sampling represents the average exposure to ethanol during the collection of the urine in the bladder.

Urine ethanol testing techniques include those used for blood-sample testing, such as gas chromatography. However, alternatives exist, such as the use of EtG tests [58], determining the level of ethanol in urine based on a color change of a strip. Once again, this method cannot identify the level of intoxication due to ethanol, but rather an estimate of exposure levels to ethanol in a specific time period. Regardless, urine testing can be used for monitoring a subject’s average exposure. Urine samples may also be used to determine the presence of other intoxicants and performance-enhancing drugs.

Besides the use of gas chromatography, urine samples may also be tested using headspace gas chromatography [59], due to its precision and the ability to distinguish aldehydes from ketones in urine. It is also key to note that urine sampling is subject to false positives, resulting from sugar consumption that when fermented in the bladder turns into ethanol. Therefore, urine sampling finds the majority of its application in general alcohol exposure testing, such as monitoring of recovering people with AUD [60].

Another alternative bodily fluid that can be used to estimate BAC is saliva. Since ethanol is highly soluble in water, its level can be estimated from saliva samples; however, this technique also comes with many shortcomings. Similar to blood and urine sampling, the techniques used in measurement of saliva alcohol concentration (SAC) include gas chromatography and enzymatic oxidation of ethanol through alcohol dehydrogenase. SAC tests find most of their applications in testing trauma patients in emergency rooms [61]. In order to perform these tests, a volume of 2 mL of saliva is required, which may be difficult to obtain from patients who are highly intoxicated or have dry mucous membranes. Moreover, saliva testing can only capture a maximum intoxication level of 350 mg/dL, and due to its location site for the sample testing, the results are prone to false positives as a result of the residue of alcohol from consumption or from oral hygiene products. Saliva testing is more useful and more accurate than urine testing, but still far from the true level of ethanol given by blood-sample testing.

**Figure 2:**
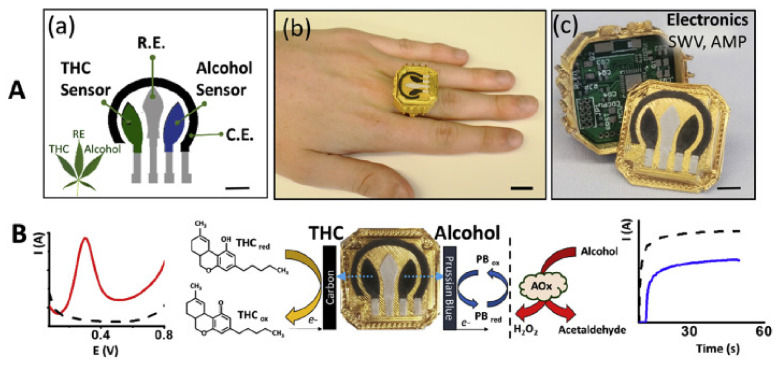
(**A**) (a) Sensor Design, (b) Real image of the sensor, (c) Ring polymeric case with integrated electronics and replicable electrodes; (**B**) voltammograms of THC detection and THC oxidation mechanism (red), amperogram of alcohol detection (blue) and ethanol oxidation mechanism.

Bodily fluid testing has not been limited to chemical color changes or analytical measurements of mixture composition. Recently developed by Mishra et al. [62] a saliva testing ring can establish the presence of alcohol and delta-9-tetrahydrocannabinol. The integrated sensors are located in the center of the ring. The system operates on the principle of electrochemical interaction between the electrodes and the substances of interest. The sensor element consists of four electrodes: two working electrodes—carbon modified with 1% MWCNT and carbon/Persian blue. The other two electrodes are the references Ag/Cl and a carbon counter. The electrode pairs are responsible for sensing THC and ethanol, respectively. The alcohol detection is established by the oxidation reaction between the ethanol molecules and the Persian blue electrode. The generated current is then used to establish the quantity of ethanol in saliva. The sensor is still in early development and requires in vivo testing. Currently, the sensors are capable of distinguishing between levels of alcohol saliva in the range of 0–0.062%. An implementation of alcohol sensing into wearable devices is mostly dominated by transdermal sensors, with few consumer-oriented products employing saliva for intoxication sensing. The above article illustrates that the development in the chemical sensing techniques is prominent and has taken advantage of the electronic system and minimization potentials, allowing for the entire device to fit inside a ring. The ring also contains a Bluetooth module for wireless data transfer. The design of the sensor and the general operation of the transducer is shown in Figure 2 below.

Amongst some of the less popular methods of alcohol testing in bodily fluids includes testing of the breast milk. The aim of testing alcohol content in breast milk is to inform the mother of an infant if their breast milk is fit for consumption. It has been shown that infants consume less of their mother’s milk following an intake of alcohol by their mother [63]. Reduction in milk consumption by an infant could also lead to nutrient deficiency in an infant. As with any other bodily fluid, breast milk may be examined using gas chromatography; however, this approach is uncommon. Retail breast alcohol testing strips are available, working on the same principle as EtG strips. Although this method allows for quick and easy testing of breast milk samples, the relationship between BAC and breast-milk alcohol level cannot be directly established. The most common type of breast-milk alcohol testing strips, produced by UpSpring, requires two minutes of submerging, after which the alcohol content reading is displayed as the change in color of the test strip, with the low threshold of 13.1 mg/dL [64]. This figure is due to variation depending on the manufacturer. A validation study by Fagani et al. [65] confirmed these findings and determined the usability of strip tests (Milkscreen) for domestic breast-milk testing. They found that the strip tests produced no false positives due to formaldehyde residues. The strip tests also showed fast results and a good level of sensitivity, similar to that exhibited by UpSpring strip tests.

Bodily fluid testing is an area of ethanol testing that produces mostly mixed results, depending on the fluid used. By far, the most accurate method of ethanol intoxication is blood testing, which is considered the gold standard of ethanol testing. On the other hand, urine provides a good enough indication of the consumption habits of alcohol, and saliva samples give good estimates of BAC with a correlation between 0.879 and 0.957 when tested against serum alcohol level [66], yet still lower than those of BrAC devices. In conclusion, bodily fluid tests for saliva and urine still have a long way to go to being used as a reliable and accurate form of measuring ethanol intoxication levels.

#### 3.3.1. Advantages

- Blood headspace GC is the most accurate method of measuring ethanol levels in the blood—Gold standard
- Small sample volume required for testing
- Storage of samples allows for delayed analysis without compromising the sample quality
- Sample kits for urine, saliva, and breast milk are readily available in retail outlets at an affordable price
- Urine and breast-milk tests yield representative results in a short time (1.5–2 h)
- The patient needs to be conscious to collect the samples.

#### 3.3.2. Disadvantages

- Blood testing is an invasive procedure, risking infection
- Very expensive equipment, specifically the GC
- The accuracy of saliva and breast-milk samples is questionable in terms of real-time ethanol levels
- Alcohol detected in urine samples represents the average exposure to ethanol during the time of urine collection in the bladder.

High consumption of sugar can result in false-positive readings in urine samples, due to fermentation of the sugar in the bladder.

### 3.4. Intoxication Estimation Algorithms

Besides the level of alcohol in breath, blood, urine, and saliva, ethanol intoxication also manifests as a change in physiological parameters and behavior. More recent developments in machine learning and image analysis have allowed for techniques to estimate the level of intoxication based on changes associated with common symptoms of alcohol intoxication. Such changes found for this review concern changes to photoplethysmography (PPG) signal, body temperature, eye position, etc. These methods provide a new outlook on measuring alcohol intoxication by not only considering the level of ethanol in the bloodstream but the degree to which a person is affected, giving rise to the true definition of intoxication as changes in behavior, regardless of the amount of alcohol ingested.

One emerging field of quantifying alcohol intoxication through physiological means is the analysis of the PPG signal, more specifically the diastolic and systolic patterns of a PPG signal, as shown by Chen [67]. Their proposed system can determine alcohol intoxication, and was shown to have accuracy of 85.71% in a study of 84 subjects. Their algorithm considers the relationship between the systolic and diastolic portions of the PPG signal and joins them with a “datum line.” This line acts as the most principal component of the correlation with alcohol intoxication. These datum lines are then stored as a dataset for supervised machine-learning algorithm (SVM) classification, although different techniques can also be used. The algorithm works by comparing small changes in the PPG signal between sober and intoxicated datasets and reaches a decision about the possible intoxication of an individual. It has been shown that a regular pattern in the PPG signal occurs when an individual ingests alcohol, and even shows a threshold after which the change in the heart rate changes significantly. As is the nature with all machine-learning algorithms, the larger the training set, the better the outcomes; therefore, future implementation of this methodology has big potential in changing the landscape of alcohol testing, whilst also outlining a fundamental physiological change that occurs as a result of alcohol intake. It is also possible that the low accuracy is not attributed to the error of the SVM classification, but rather the tolerance and individual circumstance of each of the subjects, and thus this method should be examined with much larger numbers of participants. Another criticism of this work comes from the method of validation of the results from the PPG algorithm, as per the use of BrAC device, which, as mentioned in a previous section (Breath Alcohol Devices), are notoriously associated with numerous sources of interference. The results should instead be validated using whole blood samples analyzed with gas chromatography to determine the true feasibility of this method for BAC estimation.

Development in the field of PPG analysis for intoxication levels has even been examined in comparison to ECG by Wang et al. [68]. Their study compared the operation and performance of a support-vector machine (SVM) trained for recognition of intoxication based on the features extracted from each of the respective signals. The finding of their work relates to the fact that both ECG and PPG produce identical classification performance. This has in turn motivated their work in the direction of developing an algorithm that could be applied to wearable sensors, due to its fast computation, supported by the fact that the action of the cardiac muscle is affected by the presence of alcohol in the blood. Their model performed with the highest accuracy of 88%. The current system can distinguish between sober and intoxicated level (BrAC > 0.15 mg/L). It is also key to note the calibration of the system was performed with a breath alcohol device, associating their errors into the system. Ultimately the system aims to be implemented into an already existing PPG smart wearable platform.

PPG signal analysis for detection of intoxication has already been implemented into a “smart-steering” system developed by Rachakonda et al. [69]. The system incorporates several sensor types, including temperature, respiration rate, heart rate, and blood pressure. The combination of these sensors is used to establish the condition of intoxication. Overall, the system claims to be 93% accurate in detecting intoxication. The system is intended to be implemented as an add-on device to a steering system and communicate wirelessly to the car dashboard or any smart device in the car.

Clearly, PPG signal analysis offers a convenient way of establishing if an individual is intoxicated. These algorithms can be implemented into already well-established platforms of PPG data acquisition, especially wearable smart devices. Without a large enough calibration set, or special conditions implemented into the algorithms, individuals with irregular heart action could fall victim to false positives if this system was to be more broadly implemented. Association of changes in the operation of the cardiac muscle can be of great guidance for the degree of intoxication influence ethanol has on an individual. These can vary based on the regularity of consumption and tolerance.

The effects of alcohol intoxication are not strictly limited to the changes in the behavior of the heart but also of other parts of the body. Recent research conducted by Kubieck et al. [70] suggest a link between the facial temperature distribution and the alcohol intoxication level. Through adaptation of a multiregional segmentation procedure to analyze symmetries of facial regions, such as the nose and the forehead, through IR imaging, tracking the temperature changes on the individual’s face. Their hypothesis stated an expected reduction in the coldest area on the nose and the hottest intensity on the forehead to expand, hinting at a general increase in temperature of the facial region, supported by the vessel dilation after alcohol consumption [71].

Their work concluded that the multiregional segmentation is capable of distinguishing changes in the temperature of the nose and the forehead, associated with alcohol consumption. Their results showed a noticeable change of thermal region in each of the facial regions investigated, suggesting a link between facial temperature distribution and gradual alcohol intake, particularly useful for continuous monitoring and prevention of DUI. It is important to note that it is only an early stage of method development, only pointing at areas of consideration for IR image intoxication measure, and further work on the classification of intoxication level is required.

Similar efforts in passive driver monitoring are also being implemented by large automobile manufacturers such as Volvo [72], proposing a system of in-vehicle cameras pointed at the drivers face to establish whether they are intoxicated or experiencing a distraction. The system proposed by Volvo operates on a principle of analyzing the facial expression of the driver and the direction they are looking towards, including the time they spent looking away from the road. The system operates by splitting into three stages, Support: a gentle reminder for the driver to keep their eyes on the road; Emergency State: warning message to the driver that they are at high risk of an accident; Emergency Stop: the vehicle changes from manual operation to in-built control to pull over and stop the car at the nearest appropriate place and alerting local authorities about the incident. Volvo implemented this system into their vehicles in 2020, with the launch of their new platform SPA2.

Such implementations of image analysis both have a potential to reduce the number of incidents caused by drunk drivers, however it is susceptible to possible exploits such as covering of the cameras or false negatives from individuals with higher-than-average tolerance to alcohol intoxication [73].

Additionally, the influence of ethanol consumption impacts the impedance of the skin, as demonstrated by Chaplik et al. [74] in their pilot study, by measuring the impedance of the skin between two groups of participants, consuming water and ethanol. Their findings showed that the strongest correlation between consumption of ethanol and body impedance was apparent for measurements between the hand and leg. Their investigation also considered hand-to-hand impedance measurement, showing no significant change in body impedance. Although moderate results with correlation coefficients r ranging between 0.22 and 0.39, and when considered the relative and initial impedance values, a correlation of 0.47 was reported. The measurement of bioimpedance was conducted through BIS and ICG thoracic measurements, determining the current path in the body, and evaluated by computing extracellular and intracellular resistances.

Bioimpedance measurement of the skin provides a unique application of estimating ethanol intoxication; as bioimpedance can be measured with capacitive and induction sensors [75,76], such they can be implemented into everyday objects, for example, chairs or even textiles [77]. However, as shown by the above, the correlation between alcohol consumption and body impedance is only significant between extremities of the body parts (hands and legs). With changes to the bioimpedance of the skin, one must consider the origin of such changes, and find attributing factors. As ethanol is metabolized in the body, the excretion of its by-products, through the skin, is a phenomenon that may be linked to the changes in skin bioimpedance.

Although not an estimation algorithm, another method of measuring intoxication and/or expose to ethanol is to investigate the chemistry of the hair and nails [78]. As the body digests ethanol, the by-product of the digestion EtG, deposits in the nails and hair of the patient. The above-mentioned study has found that high exposure to alcohol contributes to increased levels of EtG in the fingernails and hair. The study concluded that most significant differences in the concentrations of EtG were found in the nails, as opposed to hair of the patients. This suggests that nails are a good indicator for long term biomarkers of alcohol consumption.

It is key to note that amongst all the categories of ethanol intoxication sensing devices, those employing changes in the physiological states of the individual, such as PPG data analysis of skin impedance, their results can only be correlated due to the extensive use of machine learning methodologies, allowing for a variety of characterizations and categorizations from the provided data sets. If given the known parameter changes associated with intoxication, systems trained using these models expand their own library of data and hence become progressively more accurate in their measurements. Given the broad impact ethanol has on the human body, systems encompassing multiple factors as an indication of intoxication can only be established with machine-learning methods. Out of all these methodologies, it may be very soon at that advancements in big data and data aggregation will be capable of determining if a mobile phone user might be intoxicated. However, these applications of machine learning are a subject to personal data privacy laws and hence not likely to be implemented any time soon.

In conclusion, modern estimation methods spam a wide variety of fields, each of them with their own respective merits and shortcomings. It is key to note for these methods that they aim to correlate changes in behavior, physiological factors to alcohol intoxication, both short and long term, yet they are largely impractical for most applications of alcohol testing, whether it would be for roadside testing or personal monitoring. However, these methods provide an important insight into the nature and behavior of alcohol in the body, potentially providing explanations for topics of tolerance and addiction still not fully understood.

#### 3.4.1. Advantages

- Behavior analysis allows for classification of intoxicated actions or tick, independently of a sensing element
- Noninvasive method of measurement
- Possibility of incorporating into wearable devices equipped with PPG sensors
- Possibility of incorporating these systems into vehicles.

#### 3.4.2. Disadvantages

- Many of the methods only serve as estimates tracking another quantity changing with intoxication, the impact of intoxication on individual is not considered
- A room for error from false positives originating from general behavior
- Skin impedance shows low correlation results, with only strong correlation being impossible to incorporate into everyday life.

### 3.5. Transdermal Sensors

Besides considering testing the behavioral and physiological changes that occur as a result of alcohol intoxication, the metabolic rate of ethanol can also be used as an indicator to the level of intoxication experienced by an individual. A series of sensory devices have been developed over the last two decades to allow monitoring of the rate of expulsion of ethanol through the skin, by measuring the contents of the sweat through wearable or standalone devices. Wearable devices have received a lot of attention over recent decades with several review papers published covering different devices available on the market as well as those in laboratory testing. The findings of these papers were included in this section as well as the addition of other techniques for transdermal alcohol intoxication measurement.

The review by Swift et al. [79] in 1992 considered the state of contemporary methods for transdermal alcohol monitoring, as well as evaluating their reliability and potential future implementations. Their review was focused on the operation of a device developed by Giner that measured the current generated by an electrochemical cell when exposed to ethanol from the sweat. The operation of the Giner device showed promising results, with a correlation of r = 0.94 (*p* > 0.001) for a variety of doses, showing that early implementation of transdermal alcohol sensors have been successful in determining the quantity of alcohol in the blood.

The review of wearable devices for alcohol use disorder by Davis-Martin et al. [80] in 2021 outlined the most noticeable devices on the market, including SCRAM CAM, GinerWristTAS, BACtrack Skyn, Proof, Quantic Tally, iontophoretic biosensing system [81], AlcoWear, sensor-equipped smart shoes, AlcoGait, and DrinkTRAC. The review categorized the wearable transdermal alcohol sensors into three categories: Alcohol-Specific Biosensors, such as SRCAM and Giner WristTAS used for monitoring of the excreted alcohol from the surface of the skin through electrochemical techniques; Physical Activity Biosensors, AlcoWear and AlcoGait to monitor physical activity of an individual by means of gyroscopes and accelerometers, paired with a smartphone; and Emotion, Focused Biosensors, such as Empatica E4, used by Leonard et al. [82], who investigated their feasibility for use on subjects with a history of alcohol use.

Within the selection of the above devices, one device that is strictly distinctive is the iontophoretic biosensing system [81]. It is unlike the traditional methods of measuring BAC transdermally through sweat by a process of measuring the current generated by an oxidation reaction, correlated with the concentration of ethanol being released through the skin; this is mostly a passive process taking measurement over long periods of time and presenting the user with data outlining their overall intoxication curve. In the case of the iontophoretic biosensing system, the measurement is taken in a short time, providing a snapshot into the level of intoxication. The iontophoretic biosensing system operates on the principle of induced sweat by pilocarpine delivery via iontophoresis—electrical stimulation of tissue to generate sweat. Ethanol in the induced sweat is then measured amperometrically with an alcohol-oxidase enzyme and Prussian blue electrode transducer. The system also interfaces with a smartphone device for continuous monitoring of ethanol levels in the sweat on a flexible printed electronic circuit. Although limited in in-vivo testing, the results showed correlation of 0.99 between the level of current measured by the system and the BAC level. Further testing is required to verify the operation of this system and its applicability for continuous alcohol measurement in the future.

It is key to note that the review considered many works including those concerning the level of alcohol in tissue, but also those focusing on the physical and mental influences of alcohol use, to control (AUD). The review however is limited only to electrochemical wearable devices for measuring intoxication levels. This review considered two alternative techniques for measuring tissue alcohol concentration (TAC), using Enzymatic Biosensors and Biosniffers.

**Figure 3:**
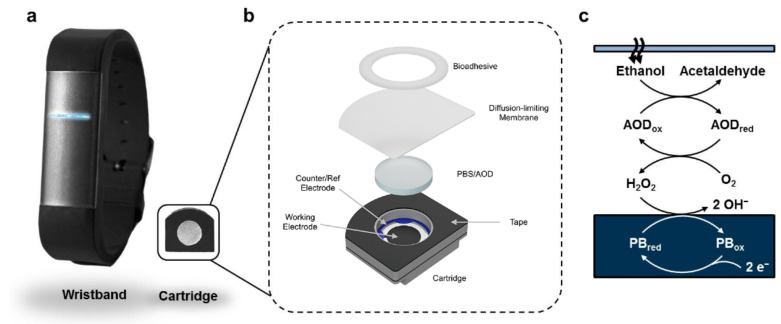
(**a**) Primary functional components of the sensor; (**b**) Primary functional components of the enzymatic alcohol sensor; (**c**) Chemical pathway for alcohol detection.

Developed by Lansdorp et al. [83] an Enzymatic Alcohol Biosensor utilizes enzyme alcohol oxidase (AOx) and a screen-printed Prussian blue (PB) electrochemical sensor as a transducer. The device operates one the prisms of replaceable cartridges, due to the denaturing of enzymes over time. The device was tested on individuals and showed a strong relationship between the BAC level and the current generated from the enzymatic biosensor. This particular method of acquiring the level of TAC is useful for continuous and long-term monitoring of subjects, especially for alcohol toxicology research. Currently the device has a US patent and is undergoing further development. The components of the system are shown in Figure 3.

Development in transdermal sensors is not exclusively limited to wearable devices, but also to standalone devices. A Biosniffer device developed by Arakawa et al. [84] based on the enzymatic reaction of alcohol dehydrogenase (ADH) targeting ethanol released through the skin as gas. The device operates on the principle of pumping a carrier gas mixing with the gas released through the skin and passing through to the Biosniffer system for detection and measurement of ethanol concentration, through fluorescence detection. Their findings concluded that gas chemistry analysis with fluorescence is a feasible way of accurately measuring concentration of ethanol in the blood and also to investigate the metabolic rate of ethanol as well as other blood gasses.

Developed by Selvam et al. [85], a wearable biochemical sensor moving away from the traditional measurement of intoxication by not measuring the ethanol concentration in the skin, but rather considers the amount of ethyl glucuronide (EtG) as a metabolite of ethanol [86]. The device operates on a principle of electrochemical sensing using gold and zinc-oxide electrodes. As EtG is a metabolite of ethanol produced by the liver, the detection of EtG from an individual’s tissue can be used to examine relapsing drinkers or confirm abstinence. As EtG stays in the body for up to 3 days, the device can also be used to take snapshot measurements of the EtG concentration in the tissue, giving it a broad area of application.

In consideration of the use of electrochemical sensors to measure ethanol metabolites, much attention must be given to the natural occurrence of the metabolite and any possible interference from associated by-products. Regardless of these interferences, the sensor presented in this paper achieved a linear relationship between the amount of EtG measured and the impedance of the electrodes, with r2 = 0.97.

Conclusively, the field of active wearable sensors is expanding. However, a distinction must be made regarding the nature of measurement. The developed sensors do not have the ability to quantify the level of intoxication, but only as an indicator of the presence of one of the metabolites of ethanol, not the BAC of a subject.

Alternative methods to measuring alcohol levels in the tissue are not limited to analyzing the sweat components, but can also be more active in their operation. An example of this was presented by Venugopal et al. [87] with a development of an interstitial fluid (ISF) sensor for alcohol monitoring. The ISF is extracted by using vacuum pressure from micropores on the stratum corneum layer of the skin. The pores on the skin are created via near-infrared laser focused on a black dye on the skin. This method is outlined to be essentially painless. The findings of this paper state that alcohol can be detected in ISF within 15 min of consumption and shows a linear relationship with the concentration of ethanol in the concentration range 0–0.2% with a resolution of 0.01% through biomedical transducers. In addition, this system is interfaced with a wireless health monitoring system linked to a wide area network (WAN) for continuous patient monitoring. Their findings also report the illumination system is only viable for three days. A prototype of this system is shown in Figure 3. This method may be considered as both transdermal alcohol sensing and bodily fluid ethanol testing due to its hybrid nature. ISF is a difficult matrix to work with, once extracted from the skin, due to evaporation [88], [89] limiting the time for analysis. ISF extractions also yields low volumes of fluid for testing. This significantly limits usability of ISF since precise analysis requires on-site testing.

More recent developments in the field of analyte sensing in ISF, implement electrochemical sensing methods, so much so, they are capable of monitoring multiple analytes simultaneously. Tehrani et al. [90], presented a technology that is capable of monitoring combinations of analytes. The two modes available are glucose and lactate and glucose and alcohol. The presented device incorporates an array of microneedles penetrating deep into the skin to access the ISF. The authors of the paper report good correlations between the measurements taken by the wearable device and correspond well with blood and breath references. However, with the breath reference mentioned, this device would require further testing, with a reference of gas chromatography for more reliable results, due to the uncertainty of breath alcohol devices, discussed at length in this paper.

The reviews concerning the current state of transdermal technology for measurement of alcohol intoxication include those by Fairbairn et al. [91], categorizing the use of transdermal sensors into four fields of application for the new-generation transdermal sensors: prevention, intervention, medical, and motor vehicle research. A systematic review by van Egmond et al. [92] evaluated the operation of transdermal devices in the field of clinical study.

In conclusion, transdermal sensors have been at the forefront of innovation in the field of noninvasive alcohol intoxication sensing, strongly supported by the number of review papers already published on this topic. Transdermal sensors provide a unique outlook on the measurement of alcohol intoxication by their incorporation into wearable systems linked with smartphones, increasing both the number of sensors available to measure intoxication but also the computing power for processing of all the data points gather from those sensors. However, both electrochemical sensors and enzymatic biosensors suffer from the wear of materials over time impacting the operation of the sensors, hence increasing the error in the measurements it gathers. This is partially mitigated by the enzymatic biosensors which incorporate a replaceable cartridge; however, this increases the engagement of the user, and introduces the possibility of human error, through damage or incorrect placement of the cartridge. Moreover, transdermal sensors for ethanol sensing can be susceptible to false positives from environmental factors, such as alcohol-containing cleaning fluids or ethanol in the air. The next generation of alcohol sensing devices should aim to move away from using systems that are directly exposed to a chemical process and instead focus on more passive forms of measurement, such as the use of light spectroscopy.

#### 3.5.1. Advantages

- Small form factors, and portable for most of the devices
- Noninvasive method, sensing ethanol in the sweat
- Monitoring the patients over long periods of time, establishing intoxication profiles
- Large potential for personal monitoring and intoxication studies applications
- Incorporated with wireless communications.

#### 3.5.2. Disadvantages

- Most of the devices available are experimental and conceptual devices, except SCRAM bracelet
- Cannot isolate the value of BAC at a specific point in time, average over time only
- Delay between absorption in the blood and release of ethanol in the sweat
- Must be worn continuously/over long periods of time to establish readings
- Enzymatic based sensors require cartridge replacement.

### 3.6. Optical Spectroscopy

The transdermal sensors described in the above section focused on the ability of the sensor to capture the release of ethanol or ethanol related compounds to estimate the BAC. These methods, however, are susceptible to the variance between individuals, both the metabolic rate of ethanol elimination and in the variance of individual’s physiology such as the rate of sweat release. Optical spectroscopy aims at eliminating the bias caused by individuals’ ability to excrete sweat, by considering not the release of gases and liquids by the skin tissue, but rather investigating the state of the tissue itself. It has been shown that optical spectroscopy can be applied to measure the concentration of an analyte to a high precision, both in transmission and diffuse reflectance modes [93]. This is supported by numerous publications related to blood oxygenation and blood glucose level monitoring [94,95,96].

Consequently, the field of using optical spectroscopy has grown over to investigating the tissue spectroscopy changes associated with alcohol consumption, not by estimating BAC via metabolic rates of ethanol and sweat release, but rather directly by measuring its concentration from tissue, through analysis of the detected light intensities, spectra shift or time delays.

Since ethanol is highly miscible in water and its presence in tissue can be clearly detected in the dermal layer of the skin, consisting of roughly 65–70% of water; therefore, the probability of finding ethanol in the tissue post consumption is very likely. Most of the problems associated with optical spectroscopy of biological analytes focuses on the selectivity (differentiate between compounds in a mixture) and the specificity (assessment of a particular compound in a mixture) of the device/model implemented.

This review focuses on a particular optical technique, of using near-infrared spectroscopy (NIRS) to detect the presence of ethanol. Besides NIRS, other optical techniques will also be evaluated.

The most notable devices for measuring TAC, are the TruTouch AlcoSense devices, including TT1100 and TT2500 [97], as the first and second devices developed respectively. Both devices operate using NIR absorption spectra to measure the skin tissue. Based on the Michaelson interferometer Fourier transform IR (FTIR), operating in the wavelength range between 1.25 and 2.50 µm, capturing the first overtones and combination bonds of C-H and O-H bonds, which are specific to the structure of ethanol, but not exclusive [98]. In the later version of the device (TT2500), an InGaAs detector was used for recording the reflected signal. Both the devices utilized diffuse reflectance mode, with TT1100 sampling from a whole hand and TT2500 sampling from a finger only.

In the US patent for TT2500 [99] the system outlined is divided into four main sections: The illumination/modulation system, consisting of VCSEL electronics, VCSEL light sources, and homogenizers; the sampling subsystem, made from an optical probe and homogenizer; the data acquisition subsystem, made of the lens and the detector crystal; and the computing subsystem consisting of elements such as data processing, display, memory and communications. TT2500 itself has three iterations—MARK I, MARK II, and MARK III—and is a standalone device for testing individuals and a system to be implemented into the ignition system of a vehicle respectively. MARK I and II devices have been, implemented in testing heavy machinery users, to ensure safe operation. The MARK III version of the device is still undergoing trials for implementation in-vehicle testing. The results obtained from TT2500 clinical trials showed high correlation between the BAC and TAC, in the case of measuring site being the finger. The results achieved when measuring at the forearm were less reliable. This could be attributed to the thickness of the skin at the forearm, but also the abundance of blood capillaries in the finger. The authors also considered the difference in probe design as an attributing factor to the difference between sites of measurement. The research regarding the analytical side of the operation of these devices was conducted by TruTouch Technologies [100,101,102,103].

Wen-fei et al. [104], also proposed a noninvasive measurement of BAC based on an NIR dynamic spectrum. The beat of the human heart causes the expanding and shrinking of blood vessels, and by using a set of dynamic spectra and extracting the fundamental frequencies using FFT, the data was compared to the original dynamic spectra (sober) and the dynamic spectra of a test subject who had imbibed a quantity of ethanol and used to estimate the BAC based on the changes in the fundamental frequencies. The results obtained showed a strong correlation between the change in frequency of the signal and the intoxication state, with an error as low as 0.6%, averaging out at 3.26%. Conclusively, their work proved that it is possible to accurately measure the BAC level for an individual, through partial least square (PLS) analysis of a dynamic spectrum.

**Figure 4:**
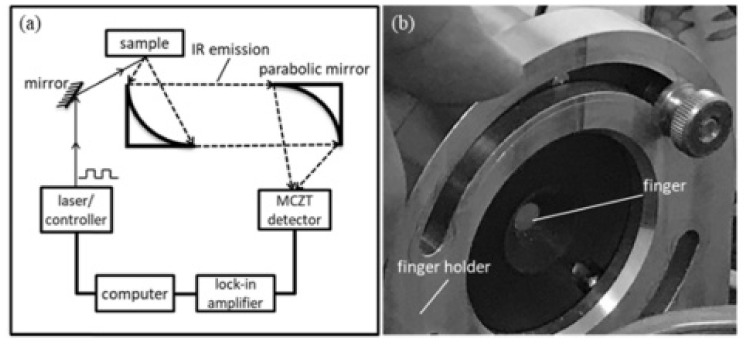
WM-DPTR system. (**a**) schematic diagram of system setup; (**b**) finger holder used for in vivo measurements [105].

Alternative to NIRS techniques, a method developed by Guo et al. [105], used wavelength modulated differential photothermal radiometry (WM-DPTR) to estimate BAC in the mid-infrared range. The WM-DPTR method is based on the modulation of laser-beams which are out-of-phase in respect to each other, where one of the wavelengths is the peak absorption and the other is the baseline for the analyte, in this case ethanol. Their study found that WM-DPTR can be used to estimate the level of BAC at a resolution of 5 mg/dL with the detection limit of 10 mg/dL. Similarly, to other optical spectroscopy devices, this technique is aimed to be implemented into the vehicle operating system as a direct preventative factor against DUI. The report also found the optimal peaks for ethanol detection in MIR as 9.56 and 9.77 µm to avoid the interference with glucose, which occupies similar frequency bands as ethanol. The schematic of the system and the measuring setup are shown in Figure 4.

Alongside the developments in the broad field of spectroscopy, a suggested system by Yamakoshi et al. [106] approaches detection of ethanol the same way as measuring oxygen saturation with pulse oximetry. Pulse alcometry is alternatively “integrated sphere finger photoplethysmography.” The technique is based on the principle of optical density, removing the baseline over the measured wavelengths using second derivatives (1150, 1185, and 1220 nm). A regression model was then implemented with the measured optical density at each wavelength to predict BAC. The developed technique has been relatively successful, achieving correlation between 0.773 and 0.846. Their system had a varied error between 0.134 and 0.333 mg/dl. The system utilizes transmission FPPG combined with an integrated sphere element. The use of the integrated sphere was justified by an enhancement of scattered light collection. However, as noted in their original work, this method still requires more research and investigation. The authors specifically mention the limitation of their work, mentioning the size of the sphere, placement of the finger, comparison with reflectance Finger-PPG (FPPG), and influences of skin color. Conclusively, this technique has plenty of potential for measurement of constituents of blood, such as ethanol. However, considerations of feasibility of application remain questionable, due to the small sample size of the pilot study.

In more recent times, Rockley Photonics have been promoting their VitalSpex biosensing platform. The parameters measured by this system include heart rate variability, respiration rate, pulse oximetry, blood pressure, core temperature, hydration. These parameters are included in the Baseline version of the VitalSpex. The extension of this platform under the name VitalSpex Pro, include additional sensing parameters, including blood alcohol content, blood lactate and glucose indicator. The expected date for this platform to become available will be some time in 2023 [107].

Unlike transdermal sensors, optical spectroscopy sensors have not seen as much interest as electrochemical or enzymatic sensors, with only a handful of published works and even fewer devices available on the market. This presents a question regarding the current state and the future of in-vivo alcohol sensing technology. It is key to note that there have been no optical spectroscopy wearable devices developed for measuring ethanol intoxication, as far as this review is concerned, presenting a unique gap for future research and development in the field. Other developments in the field of fiber optical sensors in alcohol have already been summarized by Memon et al. and by Pathak et al. in their respective reviews [108,109]. These reviews focus specifically on fiber optical based ethanol sensors.

#### 3.6.1. Advantages

- Complete noninvasive blood alcohol measurement
- No requirement for regular component replacement
- Very fast acquisition time
- Potential for simultaneous analysis of other analytes
- NIR radiation is unaffected by skin pigmentation
- Wearable device implementations.

#### 3.6.2. Disadvantages

- Mostly large devices, with high power requirements
- Very expensive to develop/purchase, specifically for TT2500
- Highly susceptible to error due to difference in skin thickness (path length)
- Reflectance spectroscopy is significantly less accurate than transmittance spectroscopy, hence wearable implementations are difficult.

## 4. Discussion

The field of measuring ethanol blood concentration encompasses a wide range of techniques, including pharmaceutical, electrochemical, enzymatic, optical, and in certain cases evaluating changes to physiological signals, such as the PPG and dynamic NIR spectroscopy to establish patterns associated with alcohol intoxication. Considerable attention is given to the application of BAC testing for prevention of DUI incidents, especially shown by the BrAC and NIRS devices [110]. Since vehicle incidents are a common occurrence for drivers under the influence of any drugs, it is obvious that an integration of an “intoxication sensor” would assist in combating this reckless behavior.

Electrochemical and enzymatic sensors are the most diverse range of innovations in the field, with a wide range of techniques, both for signal acquisition and processing. The results of this literature review also indicate their main application in long-term monitoring of individuals either in law enforcement for individuals who have a history of alcohol-related crimes or in alcohol intoxication related studies, where it is not feasible to regularly take blood or breath samples from an individual. Transdermal sensors are also particularly useful in cases where the cooperation of a subject is not possible, such that gaining a breath or blood sample is not possible. However, those sensors as previously discussed are only as good as the pharmacokinetic model on which they are based, and any variance from the model will increase the error of measurement. Transdermal sensors also present a unique opportunity to introduce personal alcohol testing by means of wearable technology. While the instrumentation required for electrochemical/enzymatic in ethanol testing can be miniaturized, the use of chemical/enzymatic sensors presents a unique shortcoming regarding the operating life of such sensors. Since they depend on either enzymatic reaction rate or deposition of by-products on electrodes the wear experienced by these components will impact their operation over longer periods of time. This can only be resolved by regularly replacing the sensing component of the device, increasing the cost to the consumer and labor manufacturing overheads.

A cheaper alternative to wearable electrochemical/enzymatic devices are the strip tests available for bodily fluid testing. These are inexpensive and simple to use; however, they are by no means an accurate method of measurement of BAC levels, but rather indicators of the presence of alcohol over a specific period of time. Although bodily fluid alcohol testing is far more advanced in the forensic field of study [111], by means of instruments such as gas chromatography, which are to be considered as the gold standard for BAC testing. This method however is expensive and requires trained staff, and in some cases an additional instrument of mass spectrometer, which are naturally very large and nonportable.

A narrow field of measuring physiological changes in the body included the systems such as the one implemented by Volvo to monitor the facial expression and the gaze for accident prevention and the measurement of changes in IR imaging of the subject’s face, gave an indication as to what changes occur in human behavior when they are under the influence. Such systems carry a unique advantage, as they are passively measuring the effects of intoxication, and can act when the effects of intoxication put lives at risk. However, as with any of the devices and the techniques reviewed in this paper, there are shortcomings. Implementation of such a system in the next generation of cars, would be challenging, and thus require a lot of capital investment. The system must also be fault proof, such that a person who just simply “looks” tiered is not recognized as a false positive. It is also important to remember that these systems do not measure the level of ethanol in a subject, instead they consider changes in the face position or surface skin temperature, and therefore the true estimation of BAC by these methods is unreliable and highly dependent on the data collected for calibration or the training of the model. However, the innovation of the automotive industry with the aim to limit the number of incidents caused by DUI is to be applauded, yet the application of a correct method is also a question worthy of exploring.

This has subsequently increased in the interest of monitoring intoxication of drivers in the car, by devices such as the one developed by Autoliv (Ljungblad et al.) to measure the exhaled air ethanol content and traces the origin of the ethanol through a CO_2_ sensor, a method like BrAC devices. Unlike the one suggested by Volvo or the thermal imaging comparison measure the presence of ethanol. But as for any BrAC device the conversion rate of 2100:1 for the BAC estimation combined with all the uncertainties of breath measurement itself demean the accuracy of these devices. Moreover, the question should also be raised about the practicality of measuring BrAC passively in a closed car, with multiple subjects where the driver may not necessarily be intoxicated but one of the passengers can possibly exhale ethanol and set off a false positive.

Therefore, a more feasible application of BrAC devices lies with the application of a breath alcohol device, where a subject has to blow into a tube, and the breath sample can be analyzed with any interference from other passengers in the vehicle. The accuracy of the BrAC devices has been proven to be effective at estimating BAC of a subject to a relatively high accuracy, yet not specific enough compared to gas chromatography. BrAC devices also depend highly on the cooperation of the subject, and the results of a BrAC test can be manipulated by controlled ventilation before the test is undertaken. However, as a deterrent factor for DUI, breath alcohol devices help to prevent many accidents every year, but only at one point in time. Implementations of BrAC devices exist, where they are linked to the ingestion system and require a driver to blow into a BrAC device before the vehicle can be engaged [112]. This, however, is not person-specific and can be tricked by a breath sample of a sober individual who is not the driver.

For any of the physiological devices/techniques, to find an appropriate implementation would need to link the system to an alcohol sensor, to justify the changes in the physiological factor that is measured. Without the establishment of ethanol consumption, these systems are only making estimates about the level of intoxication. Therefore, any of these implementations must be combined with a sensor for ethanol level, either the tissue or breath. However, physiological changes devices and techniques are useful at pointing out the outlining of the changes that may be associated with ethanol consumption. Therefore, these sensors would find a far more applicable implementation in determining the physical state of an individual to assess whether they are physically impaired by ethanol, as the variance in ethanol metabolism and tolerance is high. These devices would be more appropriate in determining the physical impact of alcohol intoxication and to determine the state of the person, rather than trying to establish an estimate of BAC, which would reach a bottleneck for subjects consuming high doses of alcohol.

Ultimately, all techniques reviewed in this paper show that the effects of ethanol intoxication can be attributed to many changes in physiological behaviors, yet the results they obtain for some of the techniques are mostly insignificant or are not feasible for everyday applications. These reasons may include the time required for measurements or the principle of measurement that may be faulty by not considering other compounds which originate or increase from their normal concentration during an intoxication period. In the case of devices which measure physiological changes, the measurement is only noted as a change of a physiological factor, which may not be an exclusive change for ethanol intoxication. These types of devices and techniques are most likely not going to find any application for real life intoxication testing, since the knowledge of intoxication is given in all of them, and they are not well adapted for recognition of false positives, such as tiredness or use of medication with similar effects as ethanol. However, one must also consider the fact that none of the devices and techniques reviewed in this paper considered the use of neural networks or any other deep-learning algorithms for classification of data, A system that would encompass all three of these fields would be the new competitor in the field of ethanol intoxication testing.

**Figure 5:**
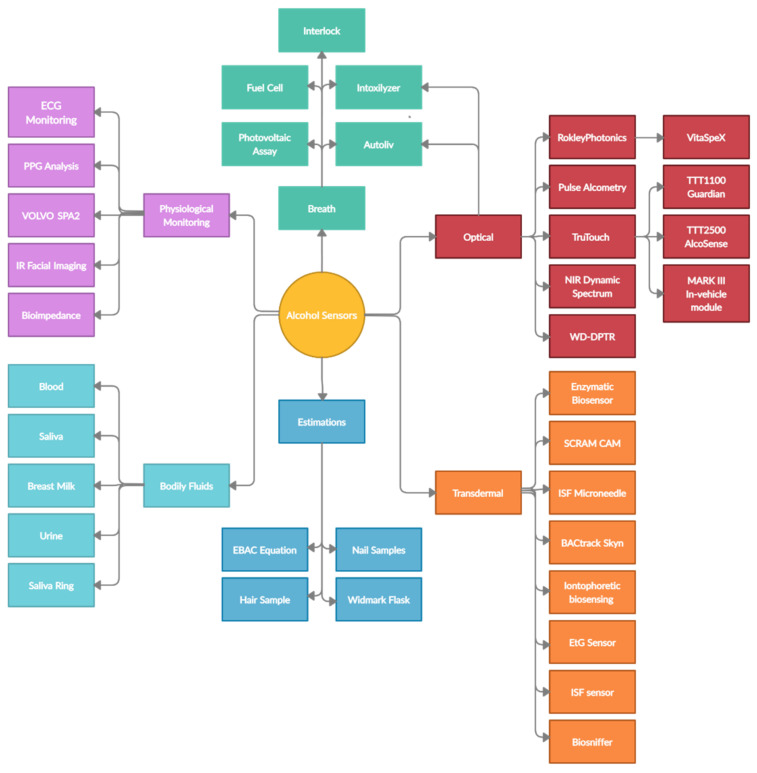
Alcohol sensing technologies by type of measurement.

Figure 5 illustrates the visual representation of the variety of techniques for measuring intoxication. It is clear to see that one of the most common methodologies of assessing alcohol intoxication is through transdermal and breath methods, with the greatest variety of techniques presented. A key point to make regarding optical technology, which is implemented not only for skin surface measurements but also applied in breath sample analysis. This link presents that optical technologies are becoming increasingly utilized for ethanol measurements both in tissue and in breath. Optical methods prove to be very effective in ethanol measurements in multiple types of media suggesting their application cover more than one field of sensing, due to their high specificity for ethanol by also their nondestructive properties.

### Future Prospects of Intoxication Sensing Technology

Considering the scope of the field of alcohol intoxication sensing, it is clear to see that the market is heavily dominated by BrAC devices, this is particularly evident in the implementations of in-vehicle passive and active alcohol detecting systems. In fact most of the developments in the field of noninvasive ethanol sensing is aimed at implementing alcohol/intoxication state in cars, with an objective of reducing the fatality rate by drunk driving. Systems which lock the driver out of their vehicle, could be of great assistance in assisting roadside safety, however they would not be amongst the desired features in a car by the user. Potential fear of false positives could result in driver being locked out of their vehicles with no direct recourse. This in turn can lead to many problems for car manufacturers, in term of customer relations. Also implementing these systems into all modern cars would require a very long time, especially if older vehicles will need to be equipped with these systems. These in vehicle intoxication sensing systems, although good in nature will most likely not be appreciated by drivers and will be very expensive to implement. Alternatively, the most likely future for this system would lie in the heavy machinery and construction, replacing the already existing breath-based interlock systems.

By far the brightest future would be seen for the new generation of wearable sensors, both optical and transdermal sensors offer a large variety of methods for measuring intoxication levels, alongside other analytes such as glucose and lactate. This makes wearable devices one of the most prominent future technologies in the field of intoxication sensing. With wearable implementations of intoxication sensors, the application of such systems would be seen from personal monitoring, research of intoxication, addiction treatment or monitoring high risk individuals. Wearable devices such as SCRAM are already in use by the law enforcement for monitor high-risk individuals; however, the form factor of the device makes it very intimidating, instead smaller version of these devices could be fitted on the wrist with the same or even improved accuracy.

Nonetheless, intoxication is not the same as blood alcohol level. When considering the estimation of the physical state of an individual, factors such as tolerance play an important role in determining the degree to which an intoxicating substance has affected them. Therefore, monitoring of multiple parameters, such as PPG and behavior changes, temperature changes can play a vital role in establishing the true image of intoxication. Combined, these parameters could be used to correlate specific conditions/diets/moods to intoxication state and the rate of ethanol elimination. Hence, the use of multiple modalities and implantations of machine learning algorithms can pave the new wave of intoxication state interpretation and maybe shed some new light on the mechanism of tolerance.

Amongst the up-and-coming technologies nanomaterials have a very high potential to be the future of biosensing application. By synthesizing strings made from specific compounds, the properties of which correspond to those of the analyte, a response triggered by their interaction can be interpreted as an indicator of the analyte’s presence. These developed sensing techniques have very high selectivity and hence are highly unlikely to produce false positives. The future of VOC sensing is very bright and full of potential innovations. These developments are described by many researchers in the commonly available literature mostly through the use of semiconductor materials [113,114,115,116].

In retrospect, the field of intoxication technology is continuously progressing and innovating new methods of detecting and quantifying ethanol concentration in the blood, where it is through analysis of chemical interactions or physiological changes in the person’s body. Further research in the field of sensing will benefit the development of treatment methods and one day will be available in all wearable devices.

## 5. Conclusions

This review of the literature on ethanol testing identified several different techniques for measuring and estimating the BAC level through numerous means, including metabolic rate estimation, breath sample analysis, physiological changes, transdermal sensors and optical spectroscopy. Although many of these techniques claim to accurately and precisely measure the BAC levels, they are far from the accuracy achieved by gas chromatography–mass spectrometry. Regardless, many of the techniques outlined in this paper showed promising and impressive results for the methods they use to estimate BAC. As shown by most of the reviewed literature, the main focus of alcohol intoxication sensing is related to incidents caused by DUI ad preventing their occurrence. A large branch of alcohol intoxication sensors focuses on transdermal techniques, analyzing the presence of ethanol or ethanol-related compounds in sweat. These devices are compact and many of them implement a wearable application, such as a smart sensor linked to a smartphone. The problem associated with these devices is the question of their reliability after regular use, due to the use of deteriorating components that may give inaccurate readings if not maintained appropriately.

Although the field of alcohol intoxication is broad, it still has not been established as a social habit to measure personal intoxication during a consumption episode. This mostly arises from the impracticality of the handheld devices, or the above outlined shortcomings of electrochemical/enzymatic biosensors. This review thus concludes that the field of alcohol intoxication measurement has not yet been introduced into the consumer domain, and current technology still has not implemented a system for using NIRS as a smart wearable device as a possible area for further innovation in this field. In addition, the measure of intoxication is not strictly limited to the amount of ethanol in the bloodstream but is also dependent on individual’s tolerance to the intoxicating effects of ethanol. Such impacts can be observed in variations such as changes in PPG morphology or face heat signatures Therefore, a combination of measurements from both the concentration of ethanol in the blood and the changes in physiological signals may be a better indicator as to the state of an individual. A device attempting this should consider individual such factors as weight, sex, and the metabolic rate of ethanol breakdown, as outlined by Widmark. By also considering recent interest in the role of other metabolites of ethanol, namely acetaldehyde and acetic acid, it is possible that the next generation of intoxication sensors will increase the number of factors. As the field of intoxication is vast and ethanol intoxication remains an intensively studied research area, so do the methods and tests used for measuring and quantifying intoxication.

## Figures and Tables

**Table 1:** Summary of ethanol detection devices and techniques.

No.	Device/Technique	Parameter	Type	Form Factor
1	Nicloux Flask	Chemical reaction	Bodily fluid testing	Flask/blood extraction
2	Widmark Flask	Chemical reaction	Bodily fluid testing	Flask/blood extraction
3	EBAC Equation	Estimation based on physiological factors	Early estimation method	Equation
4	Photovoltaic Assay	Color change based on oxidation level	Breath alcohol	Portable device
5	Intoxilyzer	Near-infrared spectroscopy	Breath alcohol	Benchtop device
6	Fuel-Cell Analyzer	Current generated by ethanol oxidation	Breath alcohol	Portable device
7	Semiconductor Breath Analyzer	Strip color change	Breath alcohol	Portable device
8	Ignition Interlock Breath Analyzer	Alcohol oxidation reaction—fuel cell	Breath alcohol	Portable device
9	Gas Chromatography	Evaporation and separation of components	Bodily fluid testing	Benchtop device
10	Headspace Gas Chromatography	Evaporation and separation of components	Bodily fluid testing	Benchtop device
11	Enzymatic Blood Testing	Strip color change	Modern estimation method	Strip test
12	EtG Test	Strip color change	Modern estimation method	Strip test
13	PPG Datum Line	Changes in PPG signal—systolic and diastolic	Physiological factor analysis	PPG analysis/modern estimation method
14	Face Heat-Map Distribution	IR image analysis of the forehead and nose	Physiological factor analysis	IR in-vehicle cameras
15	Volvo SPA2 Platform	Head position	Physiological factor analysis	In-vehicle cameras
16	Bioimpedance Spectroscopy	Impedance across the body, legs, and arms	Transdermal sensor	Experimental device/benchtop
17	SCRAM CAM	Alcohol in sweat	Transdermal sensor	Wristband
18	GinerWrist TAS	Alcohol in sweat	Transdermal sensor	Wristband
19	BACtrack Skyn	Alcohol in sweat	Transdermal sensor	Wristband
20	Proof	Alcohol in sweat	Transdermal sensor	Wristband
21	Quantic Tally	Alcohol in sweat	Transdermal sensor	Wristband
22	Iontophoretic Biosensing System	Stimulated emittance of ethanol from the skin	Transdermal sensor	Tattoo sticker
23	Enzymatic Biosensors	Enzymatic redox reaction	Transdermal sensors	Transdermal sensors
24	Biosniffer	Inert gas and fluorescence	Transdermal sensors	Benchtop device
25	EtG Sensor	By-product of ethanol metabolism	Transdermal sensor	Wristband
26	ISF Sensor	Extraction of ISF	Transdermal sensor	Wristband
27	ISF Microneedle Sensor	Sensing of ethanol in the ISF	Transdermal sensors	Skin-attachable patch
28	TTT1100	Spectroscopic measurement of tissue	Optical tissue spectroscopy	Benchtop
29	TTT2500	Spectroscopic measurement of tissue	Optical tissue spectroscopy	Benchtop
30	NIR Dynamic Spectrum	Spectroscopic measurement of tissue/physiological parameter	Optical tissue spectroscopy	Algorithm
31	Autoliv	Spectroscopic measurement of exhaled air	Optical breath spectroscopy	In-vehicle module
32	WD-DPTR	Spectroscopic measurement of tissue	Optical tissue spectroscopy	Benchtop device
33	Pulse Alcometry	Absorption of light at specific wavelengths and pulse variation	Optical tissue spectroscopy	PPG adaptation
34	THC and Alcohol Saliva Sensor	Saliva content reaction with electrodes	Bodily fluid testing	Ring
35	Breast-Milk Sensing	Strip color change	Bodily fluid testing	Strip test
36	Rockley Photonics VitaSpex Pro	Spectroscopic measurement of tissue	Optical tissue spectroscopy	Wristband
37	Hair Analysis	Detection of EtG and EtPA	Modern estimation method	Laboratory test
38	Nail Analysis	Detection of EtG and EtPA	Modern estimation method	Laboratory test

**Table 2:** Performance of the most notable experimental devices and techniques.

Author	Device/Technique	Year	Performance Summary	Reference
Widmark E.M.P.	Widmark flask	1918	First direct measure of ethanol blood concentrations	Early BrAC methods
Widmark E.M.P. et al.	EBAC equation	1924	Largely inaccurate by modern standards, error in the ranges of ±20% from true value	Widmark Flask and early BrAC methods
Brokenstein R.F. et al.	Breathalyzer (photovoltaic assay)	1961	Revolutionary device in the field of portable testing devices for intoxication, susceptible to environmental error and variance in lung volume across the population	Analysis of blood and bodily fluids
Mishra et al.	THC and ethanol saliva sensing ring	2020	Detection range: 0.1 to 1 mM (0.1 mM increments RSD of 1.5% (*n* = 5) Stable multianalyte sensing (THC)	Commercial BrAC device
Chen et al.	PPG datum line analysis	2018	85% identification rate 18 ms processing and identification time	Commercial BrAC device
Wang et al.	ECG and PPG analysis	2017	95% identification rate Only identifies if a subject is above 0.15 mg/dL	Commercial BrAC device
Rachakonda et al.	Multisensory steering wheel	2020	Detection between sober and intoxicated at 0.08 mg/dL Accuracy of 93%	No reference stated
Kubieck et al.	IR facial imaging	2019	No specific correlation number states Results indicate a very strong correlation between alcohol consumption and facial temperature distribution in all cases	No reference stated
Chaplik et al.	Bioimpedance spectroscopy	2019	Noticeable changes between intoxication and reference group Weak correlation with absolute impedance (r = 0.47) Sensitivity 92% Specificity 76%	Commercial BrAC device Blood-sample analysis (method unknown)
Wen-fei et al.	NIR dynamic spectrum	2011	Calibration set: R = 0.9672 Prediction set: R = 0.9384 Relative error between 0.6 and 9%, average error 3.26%	Hospital biochemical analysis
Yamakoshi et al.	Integrated sphere finger-PPG	2015	Lower SNR compared to traditional PPG acquisition method Sensitivity of 0.43 ± 0.29	No reference (Pilot Study)
Kim et al.	Iontophoretic biosensing system	2016	Correlation recorded = 0.912 High specificity for ethanol Increased accuracy of the system at higher ethanol concentrations	FDA-approved commercial BrAC device
X. Guo et al.	Wavelength-modulated differential photometry	2018	High ethanol resolution: 5–6 mg/dL Lag of 10–15 between ISF and blood ethanol Correlation between 0.96 and 0.98	Commercially available BrAC device
Lansborp et al.	Wearable enzymatic alcohol biosensor	2019	Linear sensor response between 0 and 0.05 mol/L Results of the sensor closely resemble those predicted by Widmark equation, however fall short during the decay stage, and generally underestimate ethanol readings	Widmark equation (BrAC device deemed impractical for application)
Arakawa et al.	Skin ethanol gas	2020	Strong correlation of 0.995 Range of estimation 73.9–112.1 ppb/cm^2^ Results demonstrate superiority over an ordinary biosniffer	No reference for intoxication measure stated
			Results indicate strong correlation for at least 3 distinct levels of ethanol	
Selvam at al.	EtG biochemical sensor	2016	Ethanol detection in the range of 0.001–100 ug/L Lower sensitivity at 1 ug/L with gold electrodes compared to ZnO (sensitivity of 0.001ug/L) Three distinct levels of EtG identified Correlation of 0.97	
Venugopal et al.	ISF sensor for remote continuous alcohol monitoring	2008	Generally strong correlation between 0.7203 to 0.866 Correlation between BrAc = 0.879	BrAC device and blood testing
Tehrani et al.	Microneedle ISF Lactate/Ethanol and Glucose Sensor	2022	Low cross-talk between sensing elements Correlation of 0.94	Commercially available BrAC device

**Table 3:** Commercially available devices for ethanol intoxication sensing.

Product	Stage in Development	Cost	Applications
Intoxilyzer (near-infrared spectroscopy)	Well established	High ($3.5k)	Forensic testing
Ljungblad et al. (Autoliv)	Prototypes in testing	—	Roadside safety
Urine alcohol test (strip)	Available to the general public	Low ($10–25)	Workstation monitoring
Gas chromatography	Gold standard	High ($50k)	Forensic analysis
Saliva alcohol sensing (strip)	Available to the general public	Low ($10–25)	Workstation monitoring
Headspace chromatography	Gold standard	High ($70k)	Forensic analysis
Breast-milk testing kits	Available to the general public	Low ($10–25)	Home and child well-being
Volvo SPA2	In testing	—	Roadside safety
SCRAM CAM	Generally available	Medium ($450 monthly)	High-risk individual monitoring
TT1100	Discontinued	—	Workstation monitoring
TTT2500	Commercially available	High ($300 per week)	Workstation monitoring
TT Mark III	In testing	—	Roadside safety
Rockley PhotonicsVitalSpex	First prototype release expected in 2023	—	Personal monitoring

## Data Availability

Not applicable.
